# Biomarkers for predicting type 2 diabetes development—Can metabolomics improve on existing biomarkers?

**DOI:** 10.1371/journal.pone.0177738

**Published:** 2017-07-10

**Authors:** Otto Savolainen, Björn Fagerberg, Mads Vendelbo Lind, Ann-Sofie Sandberg, Alastair B. Ross, Göran Bergström

**Affiliations:** 1 Division of Food and Nutrition Science, Department of Biology and Biological Engineering, Chalmers University of Technology, Gothenburg, Sweden; 2 Wallenberg Laboratory for Cardiovascular Research at the Center for Cardiovascular and Metabolic Research, Institute of Medicine, Sahlgrenska Academy at Gothenburg University, Gothenburg, Sweden; 3 Department of Nutrition, Exercise and Sports, Faculty of Science, University of Copenhagen, Frederiksberg, Denmark; 4 Department of Molecular and Clinical Medicine, Sahlgrenska Academy, University of Gothenburg, and Sahlgrenska University Hospital, Gothenburg, Sweden; Virgen Macarena University Hospital, School of Medicine, University of Seville, SPAIN

## Abstract

**Aim:**

The aim was to determine if metabolomics could be used to build a predictive model for type 2 diabetes (T2D) risk that would improve prediction of T2D over current risk markers.

**Methods:**

Gas chromatography-tandem mass spectrometry metabolomics was used in a nested case-control study based on a screening sample of 64-year-old Caucasian women (n = 629). Candidate metabolic markers of T2D were identified in plasma obtained at baseline and the power to predict diabetes was tested in 69 incident cases occurring during 5.5 years follow-up. The metabolomics results were used as a standalone prediction model and in combination with established T2D predictive biomarkers for building eight T2D prediction models that were compared with each other based on their sensitivity and selectivity for predicting T2D.

**Results:**

Established markers of T2D (impaired fasting glucose, impaired glucose tolerance, insulin resistance (HOMA), smoking, serum adiponectin)) alone, and in combination with metabolomics had the largest areas under the curve (AUC) (0.794 (95% confidence interval [0.738–0.850]) and 0.808 [0.749–0.867] respectively), with the standalone metabolomics model based on nine fasting plasma markers having a lower predictive power (0.657 [0.577–0.736]). Prediction based on non-blood based measures was 0.638 [0.565–0.711]).

**Conclusions:**

Established measures of T2D risk remain the best predictor of T2D risk in this population. Additional markers detected using metabolomics are likely related to these measures as they did not enhance the overall prediction in a combined model.

## Introduction

The global pandemic of type 2 diabetes (T2D) is a major challenge for health care systems globally and new tools are needed to reduce the burden of T2D. Prevention before the onset of T2D, or recognising and treating the early stages is the most cost effective way to treat T2D, before major systemic damage such as microvascular complications, retinopathy and nephropathy occur [1]. Population-based screening for T2D could be a useful tool to aid in efforts for prevention and early treatment, and number of diagnostic tools are available for early diagnosis of T2D risk, including impaired fasting glucose (IFG) test, impaired glucose tolerance (IGT) test, combined glucose tolerance (CGT) test, insulin sensitivity indexes, and anthropometric measurements. Many of these tools require a substantial investment of both patient and clinical time. New tools are needed to complement current diagnostic measures for predicting likely T2D incidence if population-based screening for T2D risk is to be carried out. Along with the well-established measures of T2D, such as fasting glucose and glucose tolerance tests, also non-glucose or insulin-related biomarkers for T2D exist, including low plasma adiponectin concentration that has been found to be a strong predictor for future T2D development [2,3]. This suggests that there is scope for establishing complementary biomarkers of T2D risk.

Emerging technologies, such as metabolomics, are increasingly popular in epidemiology [4] to capture broad based information about large sample sets. The possibility to find novel associations between disease risk and biochemical markers makes it an attractive tool not only for categorising disease states but also for finding new predictive biomarkers. Metabolomics has also proved useful in T2D research and has revealed new associations between metabolites and T2D [5], most notably that elevated branched chain amino acids (BCAA) are associated with hyperglycaemia and may be an early biomarker of T2D risk [6]. The involvement of BCAA metabolism in development of hyperglycaemia has been confirmed in several follow up studies [7,8], showing that it precedes diabetes development. Other potential metabolite biomarkers of diabetes risk include dimethylglycine (DMG) [9], 2-amino adipic acid and glycine [10,11], opening up not only new biomarker possibilities, but giving a broader view of T2D beyond those mechanisms immediately involved in glucose metabolism. Overall there is now a considerable body of research supporting metabolomics as a powerful tool for unravelling early metabolome differences related to later T2D risk [12].

We hypothesised that by using metabolomics we would find biomarkers for T2D that would improve current tools used to predict and diagnose T2D. For this we used gas chromatography-tandem mass spectrometry (GC-MS/MS) metabolomics and built a new predictive model of T2D development in a cohort of 64 year old women, and compared this with models based on existing markers of T2D risk.

## Materials and methods

### Study participants

Both studies have been carried out in accordance with the Declaration of Helsinki and were approved by the Regional Ethical Review Board in Gothenburg and all participants gave written informed consent to participate.

A population-based cohort was initiated by inviting all 64-year-old women from the Gothenburg region to participate in a screening examination for T2D [13]. The screening examination included fasting capillary whole blood glucose measurements in women with overt diabetes and repeated oral glucose tolerance tests (OGTTs) [2,13] in women without overt diabetes. The definition of IGT and diabetes were based on WHO criteria [14]. The means of the two oral glucose tolerance tests were used in the classification of IGT at baseline. From this initial screened cohort, cases with diabetes (9.5%), and impaired glucose tolerance (IGT) (14.4%) and a random sample of healthy subjects with normal glucose tolerance (NGT) were included in a nested case–control study, which included a baseline and a follow-up examination after 5.5 years. The baseline examination included anthropometry, assessment of lifestyle factors, fasting blood glucose, oral glucose tolerance test, plasma insulin and serum adiponectin [2]. With the exception of adiponectin, these examinations were also carried out at follow-up. The characteristics of the study subjects are shown in Table 1. In total, 629 women participated in the first examination and samples from 614 subjects were analysed by GC-MS/MS metabolomics (T1D n = 14, T2D n = 202; IGT, n = 203; NGT, n = 188) [2]. T1D was defined as serum GAD antibody level ≥4.6.

**Table 1:** Baseline characteristics of the 64-year-old women.

	No diabetes at follow-up		T2D (n = 202)	New T2D at follow-up (n = 69)
	NGT(n = 188)	IGT(n = 203)		
Family history of T2D, n (%)	38(19)	81(40)	67(33)	29(42)
Smoking, n (%)				
Never	79(42)	89(44)	91(45)	24(35)
Previous	73(39)	77(38)	64(32)	30(43)
Current	36(19)	37(18)	47(23)	15(22)
Alcohol consumption, g/day	8.8(8.0)	7.2(7.1)	8.3(8.8)	7.7(8.0)
Waist circumference, cm	88(9)	92(12)	97(11)	95(13)
Systolic blood pressure, mm Hg	136(16)	149(17)	151(18)	148(17)
Serum adiponectin, μg/mL	17.5(7.1)	14.1(6.7)	12.5(6.2)	12.4(6.8)
HOMA IR (pmol/L)・(mmol/L)	1.2(0.6)	1.8(1.3)	2.7(1.5)	2.2(1.4)
IGT, n (%)				48(70)
IFG, n (%)				19(28)

All values are mean(+/-standard deviation) if not otherwise stated

The re-examination was performed after 5.5 years in 500 women with diabetes (n = 159), IGT (n = 174) or NGT (n = 167) according to the classification at baseline. The remaining 129 women from the original study did not participate in the re-examination because of death (n = 23), severe disease (n = 3), because they were no longer living in the area (n = 12) or were unwilling to participate (n = 91) [2].

### GC-MS/MS metabolomics analysis

GC-MS/MS metabolomics was performed as previously described [15] using plasma extracted with methanol:water, derivitised using methoxymation followed by silylation. Samples were injected onto a Shimadzu GCMS TQ-8030 GC-MS/MS (Shimadzu Europa GmbH, Duisberg, Germany) and both scan and multiple reaction monitoring data was collected for further data analysis.

### Metabolomics data processing

A MATLAB (Mathworks, Natick, MA, USA) script and database developed at the Swedish Metabolomics Centre (Umeå, Sweden) was used for targeted analysis of the full scan data [16]. Internal standard normalisation of raw data was performed using the method of Jonsson et al [16]. Internal standard variables from each sample were scaled to unit variance and modelled together using principle components analysis (SIMCA+, Umetrics AB, Sweden) to describe overall variation in multivariate space. Vectors from the first component were then used to correct for analytical variation between samples. The multiple reaction monitoring (MRM) data was processed and normalised as described earlier [15]. The two data sets, full scan and MRM, were then merged for statistical analyses.

### Biochemical analysis

Blood glucose, insulin and adiponectin measurements were performed using standard clinical chemistry techniques at the clinical laboratory of the Sahlgrenska University Hospital, Gothenburg, as previously described [2].

### Data analysis strategy

We used baseline data in order to explore potential metabolomics markers of T2D after exclusion of medicated T2D cases and the 69 participants who developed T2D during follow-up. These subjects were used for testing of the prediction models. The candidate markers for detection of incident T2D were tested on those subjects who developed T2D during 5.5 years of follow-up. Sixty of the 69 women who developed T2D during the follow up period had IGT at baseline. The subjects with T1D at baseline were not included in the analysis.

In the screening analyses the NGT and IGT groups, excluding the 69 women who developed T2D during follow up, were compared with the T2D group in two pairwise comparisons (NGT vs. T2D and IGT vs. T2D). Logistic regression adjusted for analytical batch was used to identify potential metabolic markers. Metabolites found to be significant in both of the comparisons were considered as potential predictive metabolites. To avoid statistical overfitting of the prediction models the number of metabolites used for prediction of incident T2D was restricted to the nine that separated T2D at baseline with the highest statistical difference.

In total eight different prediction models (Table 2) were tested for detection of the subjects that developed T2D (n = 69). The rationale for constructing these models was an attempt to delineate the importance of the metabolomics markers in the present study in relation to established predictors of future T2D (see Table 2): Model 1 –based on established non-invasive data indicating increased T2D risk [17]; Model 2 –same as Model 1 and the candidate metabolomic markers in the present study (Table 3); Model 3—serum adiponectin, HOMA IR, smoking, IGT, IFG, which are well established invasive risk markers of T2D [2]; Model 4 –Model 3 and candidate metabolic markers; Model 5 –the present candidate metabolic markers; Model 6 –was developed to determine if the 9 metabolites used in the metabolomics model could be reduced with minimal loss of prediction to make an optimised metabolomics model; Model 7—serum adiponectin, HOMA IR, smoking, without prediabetic blood glucose concentrations; Model 8 –Model 6 and IFG, IGT, which are strong predictors of T2D, in order to test the suggestion that these are the strongest predictors of T2D risk in this cohort.

**Table 2:** Models used for prediction of incidence of type 2 diabetes with abbreviations and variables included in the models.

Model [ref]	Abbreviation	Variables
1: Non-invasive [2,17]	NI	waist circumference, alcohol consumption, smoking, systolic blood pressure, family history of T2D
2: Non-invasive+metabolomics	NIMet	AM + sorbitol, galacticol, mannose, galactose, uric acid, oxalic acid, glucaric acid-1,4-lactone, 3-methyl-2-oxopentanoic acid, 2-hydroxybutyric acid
3: Adiponectin [2]	AdM	serum adiponectin, HOMA IR, smoking, IGT, IFG
4: Adiponectin + metabolomics	AdMMet	AdM + sorbitol, galacticol, mannose, galactose, uric acid, oxalic acid, glucaric acid-1,4-lactone, 3-methyl-2-oxopentanoic acid, 2-hydroxybutyric acid
5: Metabolomics model	Met	sorbitol, galacticol, mannose, galactose, uric acid, oxalic acid, glucaric acid-1,4-lactone, 3-methyl-2-oxopentanoic acid, 2-hydroxybutyric acid
6: Optimized metabolomics	OMet	galacticol, mannose, galactose and 2-hydroxybutyric acid
7: Adiponectin without glucose measures	AdM2	serum adiponectin, HOMA IR, smoking
8: Optimized metabolomics with glucose measures	OMet2	IFG, IGT, galacticol, mannose, galactose and 2-hydroxybutyric acid

**Table 3:** Metabolites used in prediction of incident T2D with average P-value, false discovery rate (FDR), odds ratio (OR) and 95% confidence interval (95% CI) from the pairwise comparisons of the NGT and IGT groups to the T2D group.

Metabolite	P-value	FDR	OR	95% CI
Sorbitol	7.54E-13	1.86E-11	11.15	6.06–22.65
Galacticol	4.85E-10	7.37E-11	6.56	3.90–11.95
Mannose	3.72E-08	2.66E-08	3.89	2.52–6.35
Galactose	9.95E-06	4.56E-05	2.44	1.68–3.69
Uric acid	1.10E-08	5.20E-08	3.22	2.21–4.89
Oxalic acid	2.19E-05	2.70E-07	2.56	1.79–3.79
Glucaric acid-1,4-lactone	8.68E-07	3.29E-09	3.80	2.47–6.16
3-Methyl-2-oxopentanoic acid	4.18E-06	1.74E-05	2.96	1.90–4.98
2-Hydroxybutyric acid	3.32E-06	5.61E-05	3.40	2.17–5.75

### Statistical analysis

All statistical tests were performed using the R statistical environment (version 3.0.2). Data was checked for normal distribution and skewed variables were log transformed. Multivariate logistic regression models were used for pair-wise comparisons between the different groups (see Data Analysis Strategy). To put equal statistical weight for all metabolites, each metabolite was scaled to have a mean of zero and a standard deviation of one. Correction for multiple testing was done using the Benjamini-Hochberg method [18]. Metabolomics data results were reported as odds ratio (OR) for 1 standard deviation change in concentration of metabolite for significant metabolites. Area under the receiver operating characteristics curve (AUROC) was used to assess the discriminating power of the prediction models. AUROC was calculated with the trapezoidal rule using the R-package pROC (version 1.8). Using the same R-package the different ROC curves were tested for significant differences using a non-parametric comparison as described earlier [19]. Model reduction for the 9 significant metabolites was done by finding the optimal model using R package glmulti [20]. Akaikes information criterion (AIC) was used to evaluate goodness of fit of the models [21]. P-values corrected for false discovery rate (P_corr_) and differences between ROC curves were considered significant at P<0.05.

## Results

The characteristics of the patients included in the analysis are shown in Table 1.

### Metabolomics

The pairwise comparisons between the glucose tolerance groups found 48 metabolites that differed significantly (P_corr_<0.05) between NGT and T2D subjects and 17 when comparing subjects with IGT to subjects with T2D. The two lists were compared to find those that were significant in both comparisons, and the nine metabolites with the lowest average corrected P-values were selected for the overall metabolomics model. These metabolites were sorbitol, galacticol, mannose, galactose, uric acid, oxalic acid, glucaric acid-1,4-lactone, 3-methyl-2-oxopentanoic acid and 2-hydroxybutyric acid (Table 3), which were all positively associated with IGT and T2D.

### Prediction of type 2 diabetes incidence

To investigate if the baseline concentrations of the selected metabolites could be used for prediction of incident T2D after 5 years and/or add value to the more traditional predictors, different ROC curves were constructed and compared with each other. AUC values for the different models with 95% confidence intervals and the change to the AUC by addition of the metabolomics model (%) are presented in Table 4 and p-values for the model comparisons in Table 5. From the eight models the combination of the adiponectin model and metabolomics provided the best sensitivity and selectivity (AUC = 0.8078[0.7491–0.8666]) for detecting the cases developing T2D after 5 years. However, the increase of 1.7% in AUC when compared with the adiponectin alone model was not significant (P = 0.74) suggesting that the addition of the metabolomics set does not improve sensitivity and selectivity of the detection of the subjects at risk of developing T2D compared to the adiponectin model alone. The combination of the metabolomics model with the non-invasive model increased AUC by 9.7% (AUC = 0.6377 [0.5645–0.7108] and 0.6997 [0.6261–0.7733], respectively), though this was not significant (P = 0.24). The combination of the metabolomics model to the non-invasive model numerically increased the AUC and, thus, also sensitivity and selectivity of the prediction, though not significantly. The metabolomics model alone provides similar prediction to the non-invasive model (P = 0.73).

**Table 4:** AUC values with 95% CI for each model and the % change in AUC where applicable.

Model^2^	AUC	Change in AUC (%)^1^
1: NI	0.6377 (0.5645–0.7108)	
2: NIMet	0.6997 (0.6261–0.7733)	+9.7
3: AdM	0.7941 (0.7382–0.8500)	
4: AdMMet	0.8078 (0.7491–0.8666)	+1.7
5: Met	0.6566 (0.5771–0.7361)	
6: OMet	0.6562 (0.5789–0.7335)	-0.1
7: AdM2	0.6550 (0.5800–0.7290)	
8: OMet2	0.7760 (0.7130–0.8390)	

^1^Change in AUC after addition of metabolomics into the prediction model

^2^Model 1: Non-invasive, Model 2: Noninvasive + metabolomics, Model 3: Adiponectin, Model 4: Adiponectin + metabolomics, Model 5: Metabolomics, Model 6: Optimized metabolomics, Model 7: Adiponectin without glucose measures and Model 8: Optimized metabolomics with glucose measures

**Table 5:** Comparison of the different ROC-prediction models based on known markers of type 2 diabetes risk and those derived using metabolomics. Numbers are P-values and the ones highlighted in grey are significantly different at P<0.05.

	Model 1	Model 2	Model 3	Model 4	Model 5	Model 6	Model 7	Model 8
Model 1	x	0.2419	0.0009	0.0004	0.7313	0.7330	0.7486	0.0050
Model 2	x	x	0.0456	0.0247	0.4363	0.4248	0.4007	0.1212
Model 3	x	x	x	0.7406	0.0057	0.0047	0.0035	0.6780
Model 4	x	x	x	x	0.0028	0.0023	0.0016	0.4729
Model 5	x	x	x	x	x	0.9764	0.9733	0.2094
Model 6	x	x	x	x	x	x	0.9789	0.0184
Model 7	x	x	x	x	x	x	x	0.0147
Model 8	x	x	x	x	x	x	x	x

Model 1: Non-invasive, Model 2: Noninvasive + metabolomics, Model 3: Adiponectin, Model 4: Adiponectin + metabolomics, Model 5: Metabolomics, Model 6: Optimized metabolomics, Model 7: Adiponectin without glucose measures and Model 8: Optimized metabolomics with glucose measures

To further evaluate if the variables in the metabolomics model can be further reduced to simplify data acquisition we tested if optimization of the model against AIC criteria can provide a model with fewer variables and similar prediction. The optimization further reduced the number of metabolites in the model from nine to four with an AUC of 0.6562 [0.5789–0.7335] for the optimized metabolomics model, a difference of only -0.0004 (P = 0.98) compared with the original metabolomics model. This model included galacticol, mannose, galactose and 2-hydroxybutyric acid as predictors of T2D.

We further investigated if prediction of the adiponectin model was driven by the non-fasting glucose measurements and found that when IGT and IFG classifications were removed from the model its AUC decreased by 21.2% (P = 0.003). There was no difference between the adiponectin model without the glucose measures IGT and IFG when compared to the metabolomics and non-invasive models (P = 0.97 and 0.75 respectively). This led us to conclude that the glucose measures IFG and IGT were driving the prediction model. This was further explored by testing if addition of IFG and IGT in the optimized metabolomics model (OM2) can provide similar prediction to that of the original adiponectin model (AdM). There were no significant differences observed between OM2 and AdM (P = 0.68) confirming that non-fasting measures of glucose metabolism (IFG and IGT) were responsible for better prediction of T2D rather than adiponectin alone.

## Discussion

In this study we evaluated if using metabolomics can add value to the prediction of the onset of T2D over the already established T2D predictors in older Swedish women. The results found that the adiponectin model previously described for this cohort [2] was the strongest predictor of development of T2D, while the metabolomics model performed similarly to the non-invasive model, with 17% lower AUC value than the adiponectin model. The association between circulating adiponectin levels, insulin resistance and T2D is strong and has been previously found to be a good predictor of T2D [22,23]. However, when removing IFG and IGT measures from the adiponectin model, all models performed similarly for prediction of T2D, suggesting that these direct measures remain the strongest predictors of T2D risk in this cohort. This is not surprising given that diabetes stems from elevated glucose concentrations and is diagnosed based on these measures. A further observation is that there is no support for the concept that genetically low circulating adiponectin levels might be causally associated with increased risk of diabetes [22].

### Metabolomics

The objective of this work was to find predictive markers of T2D rather than look for mechanisms behind development of T2D, though these metabolites may provide information on potential mechanisms. The high number of sugars that were predictive is perhaps not so surprising given the overall effects of T2D on glucose metabolism and activation of the polyol pathway [24,25] which in the present work was seen as increased circulating levels of sorbitol, a reduction product of glucose, and galacticol, a reduction product of galactose. An advantage of applying predictive modelling to the metabolomics data is that these predictive metabolites are selected in an unbiased manner, bringing attention to potential links between some metabolites that hitherto have not been linked to the development of T2D. Such methods could later be used to build targeted methods measuring the wide range of biomarkers found to be predictive of T2D in earlier studies [26]. This could lead to a method for predicting diabetes development that accounts for a far broader range of factors beyond the well-defined symptoms of elevated fasting and post-prandial glucose concentrations.

### Comparability to earlier studies

A number of studies have used mass spectrometry based metabolomics for both discovering metabolites associated with glucose tolerance and for prediction of the disease when prospective data has been available [5,6,10,27–30]. Mass spectrometry based metabolomics have also been applied in prediction of other diseases, such as breast cancer [31], and the overall use of the method in epidemiology has been reviewed recently [4]. In the current literature studies utilizing prospective data and metabolomics AROC values for prediction of incident T2D range between 0.52 and 0.912 depending on the use of different variables and metabolomics data [10,11,28,32]. The present findings are comparable to the earlier studies and the sensitivity and selectivity of the prediction models are within the range of reported AROC values. Much of the variation in the predictions in these studies can be due to the different cohorts used for the studies as more diverse cohorts will give poorer prediction while more homogenous cohorts will have better prediction. Although previous studies have highlighted aromatic and branched chain amino acids (BCAA) to be associated with T2D development in other cohorts [6,28,33], these were not found among the nine most significant biomarkers used for prediction of T2D in this study, though the BCAA were still found to be associated with glucose tolerance.

Insulin and glucose, in combination with anthropometric and lifestyle related variables are the main diagnostic tools for T2D, and have been also used to predict the onset of T2D [5], though their use remains primarily diagnostic. Recently studies utilising metabolomics have reported additional selectivity and/or sensitivity in the prediction models of T2D after inclusion of metabolomics data in their prediction models [10,11,28], in line with our findings of metabolomics being able to add value to some of the existing prediction models. However, more advanced prediction models, such as those including measurements of diabetes status (e.g. IFG and IGT) and related compounds such as insulin and adiponectin were not significantly improved by the addition of metabolomics data. There was also no significant difference when comparing the optimized metabolomics model with the addition of IFG and IGT classification to the adiponectin model. As the diagnosis of T2D is based on fasting and 2-hour glucose, early measurements of these are by nature explanatory for later T2D. This makes prediction models complicated to build because patients could be close to the threshold of diagnosis and a simple measurement imprecision (199 mg/dl vs. 201 mg/dl) could change their status from IGT to T2D. Such small differences might have large impact on diagnosis but little impact on possible predictive biomarkers. Thus, finding the perfect prediction biomarker or perfect set of biomarkers based on metabolomics alone might be more complex than apparent at first glance and the standard classification tools, IFG and IGT, remain useful tools for T2D prediction.

### Applicability of the results

With the increasing burden of non-communicable diseases in the healthcare systems worldwide, using population-wide prediction of the onset of diseases, such as T2D, is an intriguing solution to counter the rise in T2D incidence. In this study the traditional measures of T2D, insulin and OGTT are among the strongest predictors for future T2D, which is expected as these form the basis of T2D diagnosis. We found that the use of metabolite markers determined by metabolomics could achieve similar prediction to these measures, as well as other established risk factors including adiponectin and anthropometry. In order to have useful biomarker-based prediction of T2D it is necessary to have tests that can be quickly and easily carried out to minimise cost and patient time. Prediction based on a single sample is less of a burden for both patients and clinical staff, provided it has adequate precision, and can also be applied in population based cohorts for mapping of diabetes risk in larger groups. Our finding that four metabolites detected using metabolomics can predict T2D risk at a similar rate to established measures suggests that there is a future scope for adding new measures of T2D risk. Although the method used to discover these markers is advanced, mass spectrometers are now common in clinical laboratories and methodology for measurement of the best biomarkers identified using metabolomics should not be a barrier to implementation after further validation in other cohorts. Ultimately a combination of different markers that reflect different risk factors and mechanisms behind T2D development could be used to get not only a measure of T2D risk, but also an indication of what is inducing dysregulation of glucose metabolism, which will be more instructive for clinicians treating people at risk of T2D.

The limitations of the present study are that we only studied women at the age of 64 and that the sample size of around 600 subjects, while adequate for detecting differences in glucose tolerance, there may not have been enough new T2D cases over five years for building a robust general prediction model based on metabolomics data only. Women at this age have a high and rising incidence of diabetes [34], making this an important target group for prediction of T2D. In this population diabetes is often undiagnosed for many years unless an oral glucose tolerance test is performed. In the present study the majority of cases with T2D at baseline were newly diagnosed and had no treatment that might have interfered with the metabolomics measurements. Selection of this specific population, while limiting the generalisability of the results, also removes variation due to gender and age, making the cohort suitable for exploratory work on T2D.

## Conclusions

We have established a set of metabolites that can predict T2D risk in 64-year-old women with similar precision to established predictors of T2D risk. These metabolites do not improve on current prediction models but can be used as an alternative method for screening purposes that could be more applicable for larger population based studies. The results suggest that in this population, commonly used clinical tools provide adequate prediction of future T2D. Thus, finding suitable tools to predict T2D is lesser of an issue than implementing widespread screening programmes to identify people at risk of T2D.
